# The TGFβ-miR-499a-SHKBP1 pathway induces resistance to EGFR inhibitors in osteosarcoma cancer stem cell-like cells

**DOI:** 10.1186/s13046-019-1195-y

**Published:** 2019-05-28

**Authors:** Tian Wang, Dexing Wang, Lian Zhang, Ping Yang, Jing Wang, Qi Liu, Fei Yan, Feng Lin

**Affiliations:** Department of Oncology, The Eighth People’s Hospital of Shanghai, No. 8 Caobao Road, Xuhui District, Shanghai, 200233 People’s Republic of China

**Keywords:** TGFβ, Epithelial-to-mesenchymal transition, miR-499a, SHKBP1, EGFR, Osteosarcoma stem cell-like cells

## Abstract

**Background/aims:**

A novel paradigm in tumor biology suggests that osteosarcoma (OS) chemo-resistance is driven by osteosarcoma stem cell-like cells (OSCs). As the sensitivity of only a few tumors to epidermal growth factor receptor (EGFR) tyrosine kinase inhibitors (TKIs) can be explained by the presence of EGFR tyrosine kinase (TK) domain mutations, there is a need to elucidate mechanisms of resistance to EGFR-targeted therapies in OS that do not harbor TK sensitizing mutations to develop new strategies to circumvent resistance to EGFR inhibitors.

**Methods:**

As a measure of the characters of OSCs, serum-free cultivation, cell viability test with erlotinib, and serial transplantation in vivo was used. Western blot assays were used to detect the association between erlotinib resistance and transforming growth factor beta (TGFβ)-induced epithelial-to-mesenchymal transition (EMT) progression. By using TaqMan qPCR miRNA array, online prediction software, luciferase reporter assays and western blot analysis, we further elucidated the mechanisms.

**Results:**

Here, CD166^+^ cells are found in 10 out of 10 tumor samples. We characterize that CD166^+^ cells from primary OS tissues bear hallmarks of OSCs and erlotinib-resistance. TGFβ-induced EMT-associated kinase switch is demonstrated to promote erlotinib-resistance of CD166^+^ OSCs. Further mechanisms study show that TGFβ-induced EMT decreases miR-499a expression through the direct binding of Snail1/Zeb1 to miR-499a promoter. Overexpression of miR-499a in CD166^+^ OSCs inhibits TGFβ-induced erlotinib-resistance in vitro and in vivo. SHKBP1, the direct target of miR-499a, regulates EGFR activity reduction occurring concomitantly with a TGFβ-induced EMT-associated kinase switch to an AKT-activated EGFR-independent state. TGFβ-induced activation of AKT co-opts an increased SHKBP1 expression, which further regulates EGFR activity. In clinic, the ratio of the expression levels of SHKBP1 and miR-499a is highly correlated with EMT and resistance to erlotinib.

**Conclusion:**

TGFβ–miR-499a–SHKBP1 network orchestrates the EMT-associated kinase switch that induces resistance to EGFR inhibitors in CD166^+^ OSCs, implies that inhibition of TGFβ induced EMT-associated kinase switch may reverse the chemo-resistance of OSCs to EGFR inhibitors. We also suggest that an elevated SHKBP1/miR-499a ratio is a molecular signature that characterizes the erlotinib-resistant OS, which may have clinical value as a predictive biomarker.

**Electronic supplementary material:**

The online version of this article (10.1186/s13046-019-1195-y) contains supplementary material, which is available to authorized users.

## Background

Osteosarcoma (OS) is the third most common cancer in adolescence and is the most frequent form of primary bone tumors [1]. The drug resistance induced recurrence and metastasis remarkably contribute to the failure in OS therapy [2, 3]. A rising number of observations indicates that cancer stem cells (CSCs), characterized by increased tumorigenicity, self-renewal ability and multipotency, have been described contributing to tumor progression and resistance to chemo-therapy [4, 5]. In OS, existence of osteosarcoma stem cell-like cells (OSCs) may explain the high rate of tumor relapse after standard therapies. Zhang W. et, al. [6] showed that OSCs results in a critical advantage when establishing lung macro-metastases. Crasto et al. [7] used disulfiram as an ALDH inhibitor to target the CSC subpopulation in a murine osteosarcoma model. They found that a reduction in metastatic tumor burden after treatment, which was comparable to that achieved with doxorubicin chemotherapy. Therefore, therapeutics targeting OSCs may be an effective strategy to overcome chemo-resistance and preventing distant metastasis.

As the heterogeneity of osteosarcoma, rather than targeting OS cells themselves, targeting the key actors secreted in the microenvironment, such as transforming growth factor beta (TGFβ), supplies an alternative approach to overcome OSCs. Studies have shown that overexpression of TGFβ is a hallmark of many cancers, including OS [8, 9]. In OS, increase TGFβ level is associated with high-grade and metastases [9]. Furthermore, studies demonstrated that TGFβ triggered epithelial-to-mesenchymal transition (EMT), tumor-initiating cell stemness, metastasis and invasion [10–13]. However, few studies uncovering the molecular mechanism underlying TGFβ-induced EMT to affect the chemo-resistance of OSCs.

The resistance or sensitivity of some tumors to epidermal growth factor receptor (EGFR) inhibitors can be explained by the presence of mutations in the EGFR TK domain [14]. However, such mutations are rare in tumors other than non–small cell lung carcinoma (NSCLC) [15, 16]. Thus, there is an urgent need to elucidate the precise molecular mechanisms underlying resistance to EGFR-targeted TKIs to provide treatment selectively to those patients who do not harbor EGFR mutations but will nonetheless respond to TKIs. In OS, EGFR has been found to be overexpressed [17] and in vitro studies have reported the expression of EGFR as well as effective inhibition of OS growth by EGFR inhibitors [18, 19], but few activating mutations in the EGFR TK domain in OS cells [16, 19]. At the meantime, epithelial tumor cells are more sensitive to EGFR inhibitors than tumor cells that acquired mesenchymal characteristics [20]. In multiple types of tumors, like OS, the response to EGFR-targeted agents is inversely correlated with EMT [21]. It suggests that OSCs and EMT are the common denominator resistant to EGFR inhibitors. Nevertheless, the precise molecular link between EMT and EGFR inhibitors in OSCs remain unknown.

Here, CD166^+^ cells are found in 10 out of 10 tumor samples. We characterize that CD166^+^ cells from primary OS tissues bear hallmarks of OSCs and erlotinib-resistance. TGFβ-induced EMT-associated kinase switch is demonstrated to promote erlotinib-resistance of CD166^+^ OSCs. Further mechanisms study show that TGFβ-induced EMT decreases miR-499a expression through the direct binding of Snail1/Zeb1 to miR-499a promoter. Overexpression of miR-499a in CD166^+^ OSCs inhibits TGFβ-induced erlotinib-resistance in vitro and in vivo. SHKBP1, the direct target of miR-499a, regulates EGFR activity reduction occurring concomitantly with a TGFβ-induced EMT-associated kinase switch to an AKT-activated EGFR-independent state. TGFβ-induced activation of AKT co-opts an increased SHKBP1 expression, which further regulates EGFR activity. In clinic, the ratio of the expression levels of SHKBP1 and miR-499a is highly correlated with EMT and resistance to erlotinib.

## Materials and methods

### Sample collection

Ten primary osteosarcoma tissue samples were obtained from a consecutive series of consenting patients according to the approval of The Eighth People’s Hospital of Shanghai from January 2013 to June 2018. All patients were diagnosed with primary osteosarcoma and did not show other tumor occurrences.

### Mutation analysis of the EGFR TK domain

Genomic DNA was extracted from CD166^+^ cells from 10 fresh osteosarcoma tumors by a High Pure PCR template Preparation kit (Roche, Mannheim, Germany). PCR and real-time amplification monitoring of peptide nucleic acid PCR clamping were performed using a CFX 96 system (Bio-Rad). Genomic DNA was used to amplify EGFR exons 18, 19, 20, and 21 using primer sequences: Ex18-F, 5′-AGGTGACCCTTGTCTCTGTG-3’ and Ex18-R, 5′-CCTGTGCCAGGGACCTTAC-3′ for exon 18; Ex19-F, 5′-CATGTGGCACCATCTCACAA-3’and Ex19-R, 5′-CCCACACAGCAAAGCAGAA-3′ for exon 19; Ex20-F, 5′-ATCGCATTCATGCGTCTTC-3′ and Ex20-R, 5′-GTCTTTGTGTTCCCGGACAT-3′ for exon 20; Ex21-F, 5′-CCTCACAGCAGGGTCTTCTC-3′ and Ex21-R, 5′-GGAAAATGCTGGCTGACCTA-3′ for exon 21. PCR products were purified using the Qiaex II gel extraction kit (Qiagen, Valencia, Calif) or Microcon-100 purification columns (Millipore, Billerica, Mass). Purified DNA was then sequenced in both the sense and antisense directions on all cases by Big Dye Terminator (v.3) Chemistry using PCR primers on Applied Biosystem 3700 DNA Analyzers.

### Sphere formation and propagation

Solid osteosarcoma tissues were finely minced and washed in DMEM/F12 (Gibico Invitrogen, USA). Then they were incubated with Accumax 1X (Innovative Cell Technologies, USA) for 30 min at 37 °C. Single-cell suspension was obtained by filtering digested tissue. Single-cells were plated at a density of 10^4^cells in serum-free medium DMEM/F12 (Gibico Invitrogen, USA), supplemented with commercial hormone mix B27 (Gibico Invitrogen, USA), EGF (10 ng/mL; PeproTech), bFGF (10 ng/mL; PeproTech), and heparin (2 μg/mL). The medium of all the spheres was replaced with fresh growth factors twice a week until cells started to grow forming floating aggregates. Cultures were expanded by enzymatic digestion of spheres with Accumax 1X (Innovative Cell Technologies, USA), followed by re-plating of both single cells in complete fresh medium. All cells were cultured at 37 °C in a 5% CO_2_ humidified incubator.

### Flow cytometry and fluorescence-activated cell sorting (FASC)

Tumor spheres were expanded by enzymatic digestion of spheres with Accumax 1X (Innovative Cell Technologies, USA). Single-cells were incubated in staining solution containing 1% BSA with the specific antibodies at appropriate dilutions. Cells were stained with primary conjugated CD166-FITC (BD Biosciences, USA) antibodies, or corresponding isotype-matched controls. Fluorescence-activated cell sorting (FASC) was performed on cells stained with CD166-FITC (BD Biosciences, USA). Quality of sorting was monitored by flow cytometry with specific antibody for CD166-FITC.

### MTT assay

The MTT assay (Sigma Aldrich, USA) was used to determine relative cell growth every 24 h for cell growth curves. A total of 10^5^ cells/ml were plated into 96-well plates, incubated at 37 °C, and cultured overnight. 20 μl of 5 mg/ml MTT was added to the media for 4 h incubation at 37 °C. Following removal of the culture medium, the remaining crystals were dissolved in 150 μl DMSO (Sigma Aldrich, USA). Absorbance (A) was measured spectrophotometrically in a microplate reader (Bio-Rad, USA) at a wavelength of 490 nm. The curve of growth was drawn with the absorbance (A) measured spectrophotometrically in a microplate reader (Bio-Rad, USA) at a wavelength of 490 nm.

### Measurement of TGFβ in tumor cell supernatants

A total of 1X10^6^ cells were plated in media containing 0.1% FBS. Tumor cell supernatants were evaluated by ELISA (R&D Systems) to determine the amount of TGF-β expressed by 1X10^6^ cells per 24 h.

### Western-blotting

Protein extracts were resolved through 8 to 12% SDS-PAGE; transferred to nitrocellulose membranes; and probed with monoclonal antibody against EGFR, HER2, AKT, FGFR, Vimentin, E-Cadherin, GAPDH, etc. The membranes were washed and incubated with a horseradish peroxidase (HRP)-conjugated secondary antibody. The different treated cells were washed with ice-cold PBS and then lysed by protein lysate (Pierce, USA). After centrifugation at 12,000 rpm for 10 min at 4 °C, the protein concentration was measured by BCA protein assay kit (Pierce, USA). Then, all proteins were resolved on a 10% SDS denatured polyacrylamide gel and were then transferred onto a PVDF membrane (Millipore, USA). The membranes were washed and incubated with a horseradish peroxidase (HRP)-conjugated secondary antibody. Protein expression was detected and quantified using the ODYSSEY Infrared Imaging System (Li-COR Biosciences, USA).

### Quantitative real-time RT-PCR (qPCR)

Total RNA for qPCR analysis was extracted using Trizol (Invitrogen, USA), treated with DNase I (Takara, USA) to eliminate contaminating genomic DNA, and reverse-transcribed into cDNA with the Reverse Transcriptase MMLV (Takara, USA). Real time PCR was performed using a SYBR Green Reagents (Bio-Rad, USA) on the iQ5 Real-Time PCR Detection System (Bio-Rad, USA). Expression of genes with relative to β-actin was determined using the 2 ^-ΔΔCt^ method.

### Lentiviral infection

CD166^+^ cells were spin-infected with 1 ml of NC-si-RNA (Gene Copoeia, USA), *SHKBP1*-si-RNA lentiviral knockdowns packbag or miR-499a mimics (Gene Copoeia, USA) packbag. CD166^−^ cells were spin-infected with 1 ml of pcDNA3.1-NC, pcDNA3.1-Snail1 and pcDNA3.1-Zeb1 (Gene Copoeia, USA) packbag. For all systems, cells were infected with lentiviral media at a multiplicity of infection of 20, in the presence of 8 μg/mL polybrene (Sigma-Aldrich, USA) overnight in a 37 °C incubator. Stable clones were selected using puromycin. The expression of was tested by qPCR.

### Chromatin immunoprecipitation (ChIP) and promoter assays

ChIP assays were performed in CD166^−^ -hSnail-HA and CD166^−^ -hZeb1 cells. For detection of interaction between the EMT factors and the endogenous miR-499a promoters, anti-hSnail-HA (Roche Diagnostics) or anti-Zeb1 (Santa Cruz) anti-bodies or unspecific rat IgG (Jackson ImmunoResearch Laboratories) were used. Around 100 bp fragments of the miR-499a promoter sequences were amplified using the primer: F, 5′ GTTGGGTTTCAGGGCTTTG 3′, R, 5′ AGAGCAGCACCCTCCAGG 3′. As for the promoter assays, for analyses of the miR-499a promoters, pGL3 reporter plasmids containing the miR-499a (2321 to 1120 bp) regulatory sequences fused to luciferase cDNA were used. Co-transfection with b-galactosidase reporter and detection of relative luciferase units.

### In vivo studies of Tumorigenicity

All experiments were carried out with female NOD/SCID mice, 3–4 weeks old (HFK Bioscience, China). Mice were maintained at the Animal Core Facility at The Eighth People’s Hospital of Shanghai under specific pathogen-free (SPF) condition. All studies on mice were conducted in accordance with the National Institutes of Health ‘Guide for the Care and Use of Laboratory Animals’. For establishment of subcutaneously xenograft models were obtained using either 10^4^ CD166^+^ cells or 10^4^ CD166^−^ cells. Cell fragments were implanted NOD/SCID mice by trocar gauge in both flanks of nude mice (3 mice for each group). The tumor volume was calculated by the formula V (mm^3^) = 0.52 × length (mm) × width^2^ (mm^2^). About 4–5 weeks after injection, the animals were sacrificed and flank tumours were removed.

### Statistical analysis

SPSS13.0 software was used. Each experiment was performed at least three times. The data were expressed as mean ± SD, and one-way ANOVA and an unpaired Student’s t-test were used to determine the significant differences of all the results. Significances are ***, *p* < 0.001; **,*p* < 0.01; *, *p* < 0.05.

## Results

### CD166^+^ cells from primary OS tissues display stem cell-like features and erlotinib resistant

**Fig. 1:**
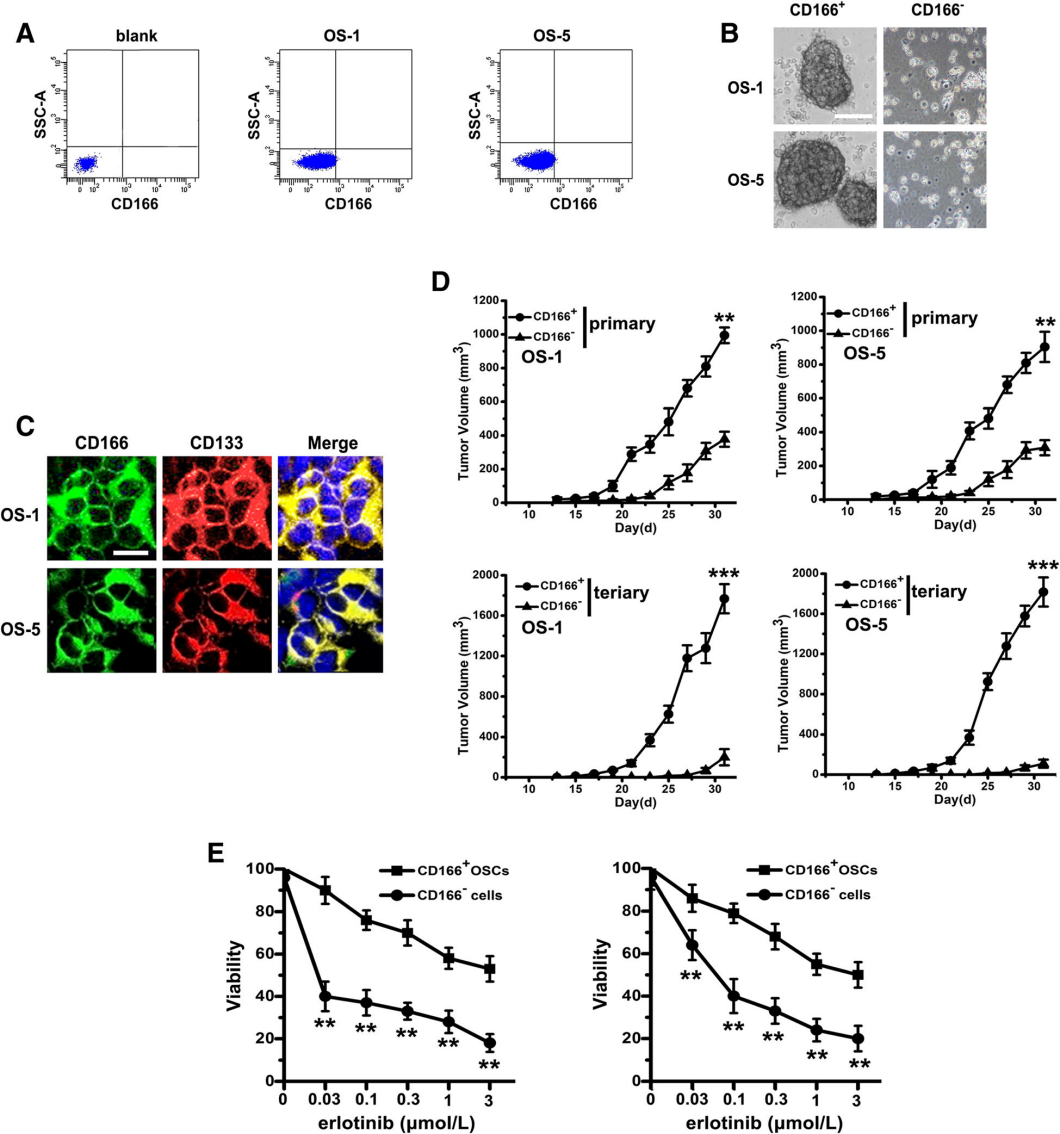
CD166^+^ cells from primary OS tissues display stem cell-like features and erlotinib resistant. **a** CD166^+^ cells were sorted from spheres by fluorescence-activated cell sorting. **b** Phase-contrast images of the ability of tumor spheres formation by seeding with CD166^+^ cells and CD166^−^ cells in serum-free medium. Scale bar, 50 μm. **c** Immunofluorescent staining of CD166 (green) and CD133 (red) expression in CD166^+^ spheres (nuclei stained with DAPI). Scale bar, 10 μm. **d** With the presence of erlotinib, cell viability of CD166^+^ OSCs and CD166^−^ cells were determined by MTT. Note: Columns, mean of three individual experiments; SD,** *P* < 0.01. **e** In vivo serial transplantation assay. A total of 10 ^4^ CD166^+^ OSCs or CD166^−^ cells from OS-1 or OS-5 in serum-free medium were injected s.c. into nude mice. Derived tumor xenografts were dissociated to single-cell suspension and then serially re-injected in mice (10 ^4^ cells), generating secondary and then tertiary tumors. Tumor growth curves of primary and tertiary tumors are shown. Note: Columns, mean of three individual experiments; SD, *** *P* < 0.001; SD,** *P* < 0.01

We analyzed 10 primary tissue samples derived from a consecutive series of OS patients. By using the surface marker CD166 alone, flow cytometry analysis showed the presence of a variable fraction of CD166^+^ cell populations in 10 out of 10 tumor samples, varying from 0.01% to a maximum of 0.47% (Fig. 1a, Additional file 1 Table S1). CSCs are believed to be able to form spheres in serum-free cultivation. Thus, we sorted both CD166^+^ and CD166^−^ cells from OS-1 and OS-5 by FACS. CD166^+^ cells were able to form compact self-renewing spheres in serum-free medium. These tumor spheres were at least passaged 10 times, indicating the self-renewable ability of these tumor spheres (data were not shown). On the other hand, CD166^−^ cells could not form compact spheres (Fig. 1b). By staining with CD133 in CD166^+^ cells, we noted that almost all of CD166^+^ cells expressed CD133 that was putative marker for CSCs (Fig. 1c).

Next, we compared the tumorigenic ability in CD166^+^ cells with CD166^−^ cells and found that both CD166^+^ and CD166^−^ cells could form xenografts. However, tumor formation of CD166^+^ cells was faster and resulted in increased tumor take compared with that observed after injection of CD166^−^ cells (Fig. 1d). By performing serial transplantation assays in nude mice of cells isolated from OS-1 and OS-5 tumor xenografts originally derived from CD166^+^ or CD166^−^ cells injection. Cells derived from CD166^+^ xenografts were able to generate tumors maintaining the original morphology and proportion of CD166^+^ cells in primary, secondary, and tertiary transplantation, whereas cells from CD166^−^ tumors lost tumorigenic potential during serial transplantations (Fig. 1d).

Certain mutations in the EGFR TK domain is one of the factors leading to the resistance to certain TKIs [14]. To determine whether such mutations are present in CD166^+^ cells, the TK domain from exons 18 to 21 was sequenced. However, we did not identify known mutations that have been associated with constitutive EGFR activation in CD166^+^ cells from these 10 primary OS tissue samples. Furthermore, by evaluating the TKIs-resistance of CD166^+^ cells, we showed that with the presence of increasing concentrations of erlotinib from 0 to 3 μM, cell viability was significantly decreased in both CD166^−^ cells compared with that in CD166^+^ cells at each concentration (Fig. 1e).

### CD166^+^ OSCs is associated with a kinase switch that enables EGFR-independent activation of AKT

**Fig. 2:**
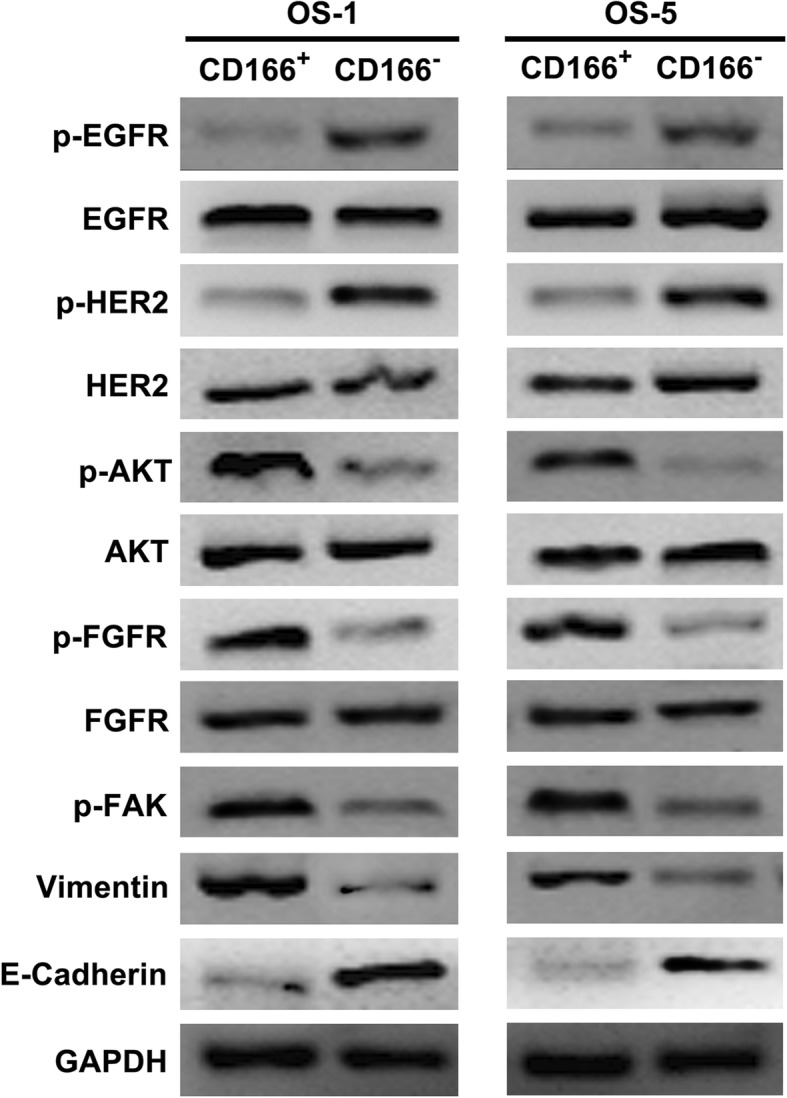
CD166^+^ OSCs is associated with a kinase switch that enables EGFR-independent activation of AKT. Western-blotting was used with antibodies specific for phosphorylated and total EGFR, HER2, AKT, FGFR, and p-FAK, Vimentin, E-Cadherin. GAPDH was used as the control

To identify the molecular mechanisms underlying the resistance of OSCs to EGFR TKI, CD166^−^ and CD166^+^ cells from OS-1 and OS-5 were used to evaluate their different response to erlotinib. We found that the levels of phosphorylated EGFR and HER2 were markedly decreased in CD166^+^ OSCs, while the expression of p-AKT was significantly increased (Fig. 2). p-FGFR level was also significantly increased (Fig. 2), which suggests that CD166^+^ OSCs exhibited a switch from EGFR to activation of an alternative tumor cell- specific RTKs (FGFR). To further detect whether resistance to erlotinib in CD166^+^ OSCs is correlated with TGFβ-induced EMT progression, we tested EMT markers and the non-receptor focal adhesion kinase (FAK) expression, which plays the key role in TGFβ-induced EMT progression. The result showed that CD166^+^ OSCs displayed a mesenchymal phenotype manifested by loss of E-cadherin and acquisition of p-FAK and Vimentin (Fig. 2).

### TGFβ induces an EMT-associated kinase switch that promotes erlotinib resistance of CD166^+^ OSCs

**Fig. 3:**
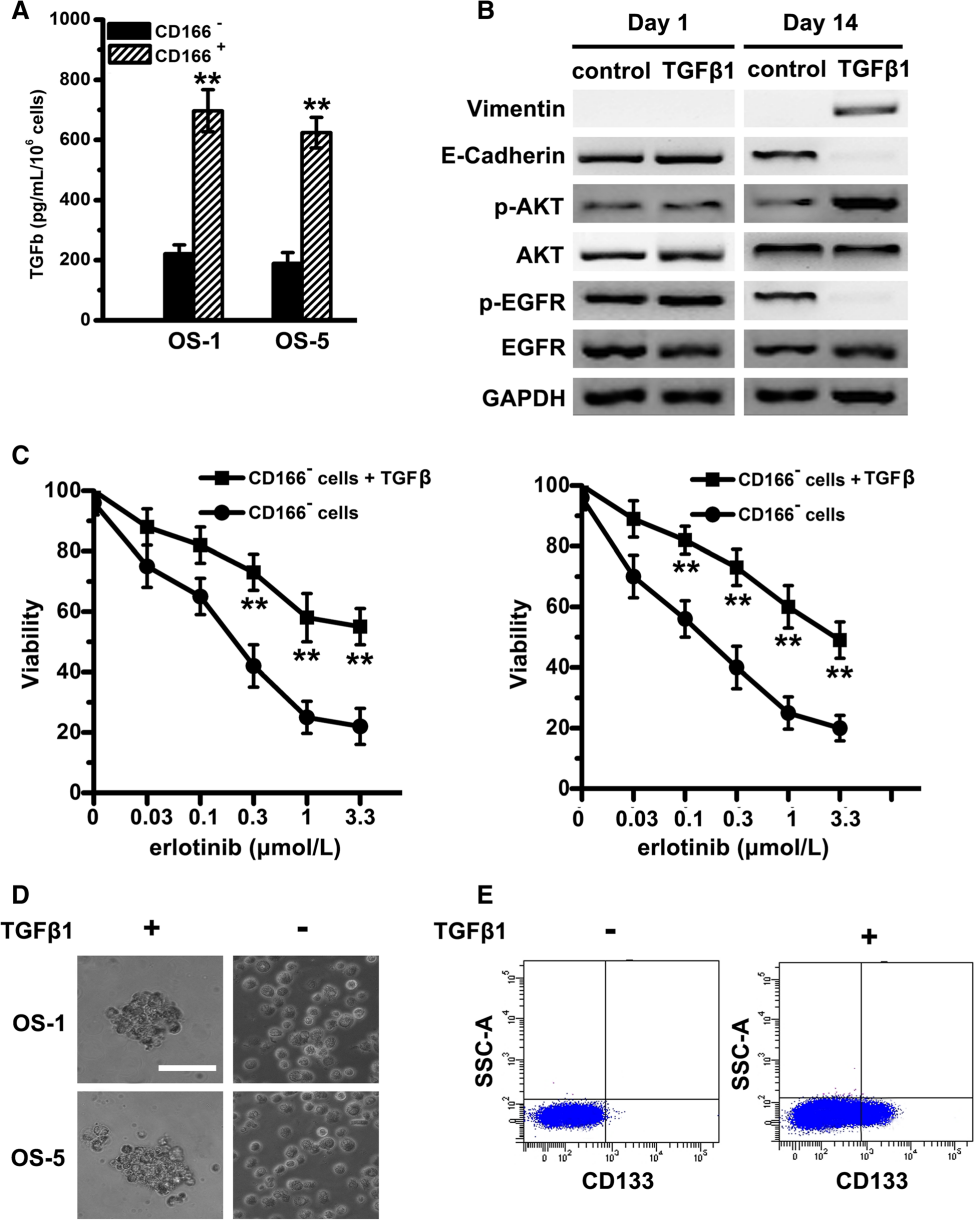
TGFβ induces an EMT-associated kinase switch that promotes erlotinib resistance of CD166^+^ OSCs. **a** Tumor cell supernatants of CD166^+^ and CD166^−^ cells from OS-1 or OS-5 were collected and differential levels of TGFβ production were analyzed by ELISA. Note: Columns, mean of three individual experiments; SD,** *P* < 0.01. **b** Immunoblot analysis was performed with antibodies against phosphorylated and total AKT, EGFR and Vimentin, E-Cadherin. GAPDH was used as the control. **c** With the presence of erlotinib, cell viability of CD166^−^ cells with or without the treatment of TGFβ1 were determined by MTT. Note: Columns, mean of three individual experiments; SD,** *P* < 0.01. **d** Phase-contrast images of the ability of tumor spheres formation by seeding with CD166^−^ cells with or without the treatment of TGFβ1 in serum-free medium. Scale bar, 50 μm. **e** Flow cytometry analysis of CD133 in CD166^−^ cells (from OS-1) without the treatment of TGFβ1

To determine whether TGFβ expression level correlates with erlotinib resistance of CD166^+^ OSCs, we measured the amount of TGFβ produced in cell supernatants of both pairs of OS cells. Compared with CD166^−^ cells, CD166^+^ OSCs secreted much higher level TGFβ (Fig. 3a). We showed that TGFβ1 treatment resulted in complete EMT by day 14: Vimentin was elevated, while E-cadherin was reduced depending on the time (Fig. 3b). At the meantime, phospho-EGFR was decreased in CD166^−^ cells with TGFβ1 treatment, nevertheless, AKT activity was significant increase (Fig. 3b), which implies that TGFβ induces the EMT-associated kinase switch. In line with these molecular alterations, CD166^−^ cells treated with TGFβ1 acquired a relative resistance to erlotinib (Fig. 3c) and cancer stem cell-like phenotype, including the ability of sphere formation (Fig. 3d) and CD133 expression (Fig. 3e).

### miRNA profiling of CD166^+^ OSCs

**Fig. 4:**
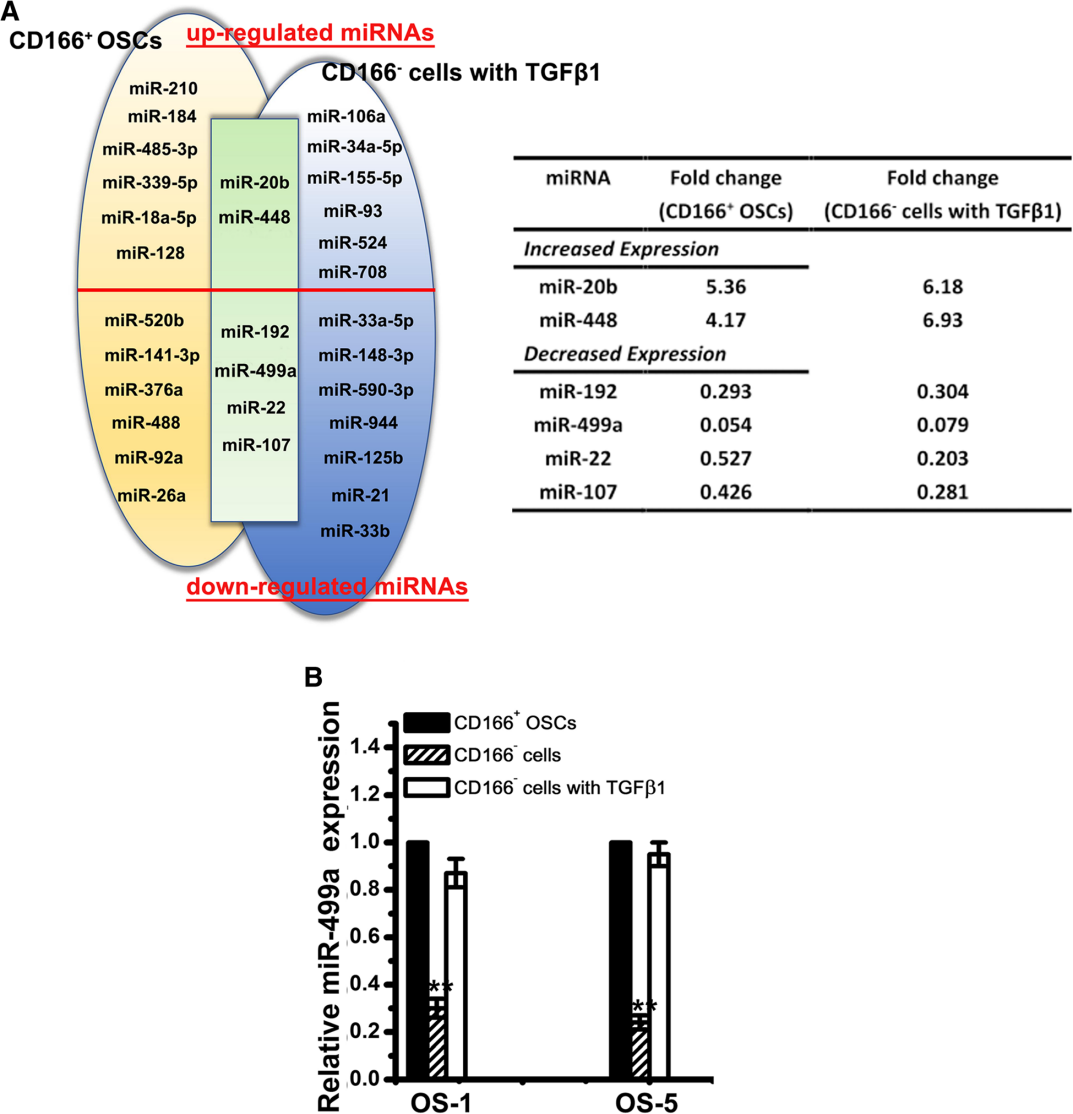
miRNA profiling of CD166^+^ OSCs. **a** Venn analyses of the up- and downregulated miRNAs in CD166^+^ OSCs from primary OS tissues (left, yellow shaded; *n* = 3) and in CD166^−^ cells with TGFβ1 (right, blue shaded), compared with CD166^−^ cells, are shown. miRNAs that were significantly and commonly deregulated in both CD166^+^ OSCs and CD166^−^ cells with TGFβ1 are shown in the overlapping area (green shaded). Only those miRNAs whose expression levels displayed greater than 2-fold decreases or increases were further studied. The table shows a summary of the significantly differentially expressed miRNAs in the overlapping area with fold change. **b** Relative expression of miR-499a in CD166^+^ OSCs, CD166^−^ cells with or without TGFβ1 from OS-1 and OS-5 were examined by qPCR. Note: Columns, mean of three individual experiments; SD,**, *P* < 0.01

To further detect the underlying mechanisms, we compared miRNAs expression levels in CD166^+^ OSCs and CD166^−^ cells with or without TGFβ1 treatment by TaqMan qPCR miRNA array. miRNAs which displayed > 2-fold changes in CD166^+^ OSCs or CD166^−^ cells with TGFβ1 were tabulated: In CD166^+^ OSCs from OS-1, 8 upregulated and 10 downregulated miRNAs were found among CD166^+^ OSCs compared with those in CD166^−^ cells. In parallel, 8 reproducibly upregulated and 11 downregulated miRNAs were identified in CD166^−^ cells with or without TGFβ1 treatment. We combined the two different data sets in a Venn diagram: 2 upregulated (miR-20b and miR-448) and 4 downregulated (miR-499a, miR-192, miR-107, miR-22) (Fig. 4a). Among these miRNAs, miR-499a was the most changed (Fig. 4a). Further validation showed that expression of the miR-499a was significantly enhanced upon TGFβ1 treatment (Fig. 4b).

### miR-499a is directly regulated at transcriptional level by Snail1 and Zeb1

To validate the direct role of Snail1 and Zeb1 in miR-499a regulation, ChIP assays were performed. Both Snail1 and Zeb1 bound to the miR-499a promoters (Additional file 1: Figure S1A). In agreement with these data, miR-499a promoter assays indicated a complete repression in Snail1- or Zeb1- CD166^−^ cells transfectants compared to the control CD166^−^ cells (Additional file 1: Figure S1B).

### miR-499a directly targets SHKBP1 expression

**Fig. 5:**
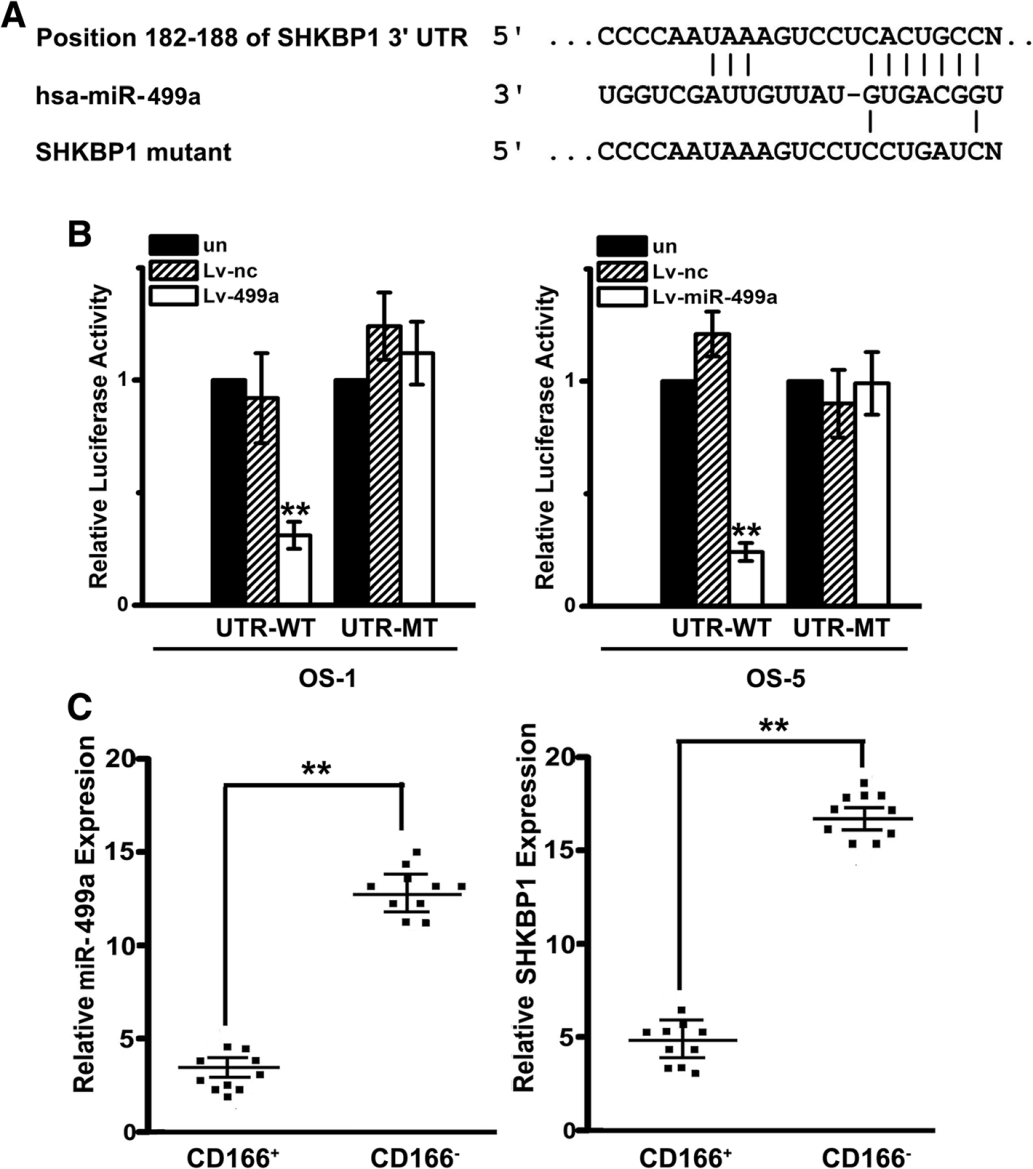
miR-499a directly targets SHKBP1 expression and SHKBP1 was negatively correlated with miR-499a level in OS tissues. **a** Illustration of SHKBP1 3’UTR as well as the seed sequence of miR-499a showed the predicted target region on the 3’UTR of SHKBP1 mRNA. **b** Dual-luciferase reporter assay with co-transfection SHKBP1 3’UTR plasmids and miR-499a mimics. The relative luciferase activity was obtained by firefly luciferase activity normalized against Renilla luciferase activity. Note: Columns, mean of three individual experiments; SD,**, *P* < 0.01. **c** Down-regulation of miR-499a was observed in OS tissues compared with that in adjacent ones by qPCR (left). Up-regulation of SHKBP1 was observed in OS tissues compared with that in adjacent ones by qPCR (right). Note: Columns, mean of three individual experiments; SD,**, *P* < 0.01

3’UTR of SHKBP1 was indicated as one of putative miR-499a targets by online prediction software, including TargetScan 6.2 and PicTar (Fig. 5a). To further confirm whether SHKBP1 is the direct target of miR-499a, luciferase reporter assays were performed on OSCs transfected with wild type or mutated SHKBP1 3′-UTR. As shown in Fig. 5b, the luciferase activity in OSCs transfected with wild type SHKBP1 3′-UTR was significantly reduced in Lv-miR-499a group. On the contrary, the effect of Lv-miR-499a was completely abrogated in the mutant construct. Moreover, to investigate the correlation of miR-499a and SHKBP1 in OS patient cohort, we detected the expression of miR-499a and SHKBP1 in 10 paired CD166^+^ OSCs and corresponding CD166^−^ cells from OS tissues by qPCR. It was shown that miR-499a levels were declined in CD166^+^ OSCs compared with those in CD166^−^ counterparts, while trend of SHKBP1 expression was contrary (Fig. 5c), which, obviously, indicated that SHKBP1 was negatively correlated with miR-499a level in OS tissues.

### TGFβ-induced EMT results in decreased miR-499a, upregulation of SHKBP1, and increased erlotinib resistance

**Fig. 6:**
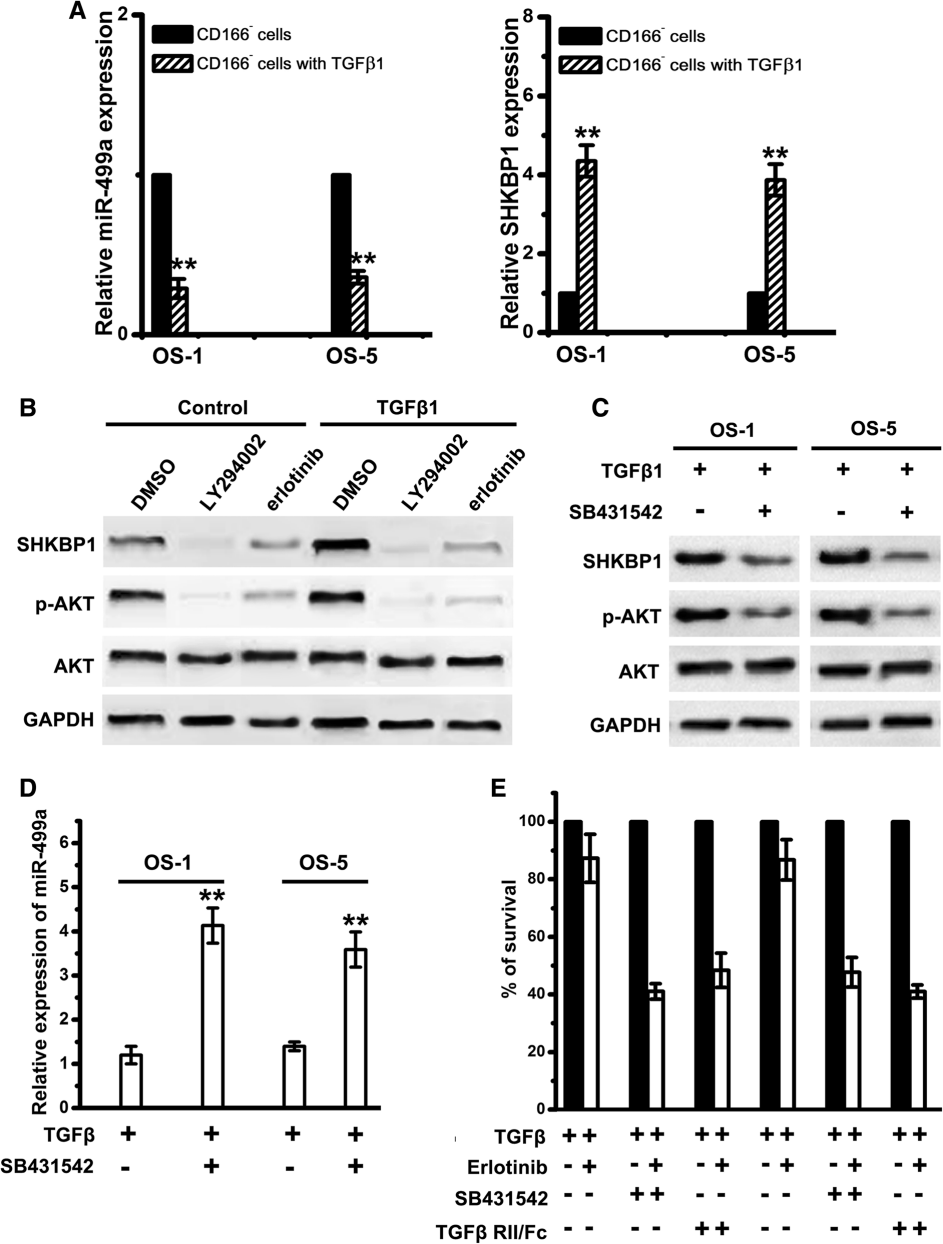
Inhibition of TGFβ signaling results in upregulation of miR-499a, decrease in SHKBP1 levels, and increased erlotinib sensitivity. **a** Relative expression of miR-499a and SHKBP1 in CD166^−^ cells with or without TGFβ1 treatment were examined by qPCR. Note: Columns, mean of three individual experiments; SD,**, *P* < 0.01. **b** CD166^+^ OSCs treated with TGFβ1 or control vehicle for 21 day were exposed to LY294002 or erlotinib for 24 h. Immunoblot analysis was performed with antibodies against SHKBP1, AKT and GAPDH. **c** CD166^+^ OSCs treated with TGFβ1 or SB431542 were used for western-blot assays with antibodies against SHKBP1, AKT and GAPDH. **d** Relative expression of miR-499a in CD166^+^ OSCs with TGFβ1 or SB431542 were examined by qPCR. Note: Columns, mean of three individual experiments; SD,**, *P* < 0.01. **e** CD166^+^ OSCs were incubated with TGFβ (2 ng/mL) alone or in combination with either SB-431542 (10 m mol/L) or TGFβ -RII/Fc (20 ng/mL) for 7 days and then were treated with 1 m mol/L of erlotinib for an additional 72 h. Cell viability was assayed and values were set at 100% for untreated controls

To test the correlation between TGFβ and miR-499a, CD166^−^ cells were exposed to TGFβ1 in a time course. We found that SHKBP1 expression was elevated with TGFβ1 treatment (Fig. 6a). As the acquisition of an erlotinib-resistant EMT phenotype in response to TGFβ was associated with a significant increase in AKT activity, we, next, detected the role of AKT in SHKBP1 upregulation. CD166^+^ OSCs were treated with erlotinib or LY294002 (PI3K inhibitor). It showed that SHKBP1 expression was upregulated in CD166^+^ OSCs (Fig. 6b). Although both of erlotinib and LY294002 reduced basal expression of SHKBP1, only LY294002 resulted in a markedly inhibition of SHKBP1 in CD166^+^ OSCs treated with TGFβ1 for 20 days (Fig. 6b). These data suggest that basal EGFR activity induces an auto-regulatory expression of SHKBP1. We, then, blocked TGFβ signalling in CD166^+^ OSCs with SB-431542, a potent inhibitor of the activin receptor-like kinase (ALK) receptors family. CD166^+^ OSCs cultured with SB-431542 showed decreased level of p-AKT and SHKBP1 (Fig. 6c), while the expression of miR-499a was markedly enhanced (Fig. 6d). Moreover, both SB-431542 and TGFβ RII/Fc (recombinant TGF β receptor II, which binds to and inhibits TGFβ1, TGFβ3, and TGFβ5) increased erlotinib sensitivity of CD166^+^ OSCs (Fig. 6e). It suggests that TGFβ-induced EMT results in the decreased miR-499a, enhanced SHKBP1 expression, and increased erlotinib resistance.

As EGFR-independent activation of the PI3K-AKT pathway has frequently been seen in cells that develop resistance and is thought to confer resistance to EGFR TKIs [22]. We further sought here to determine whether EMT-associated PI3K-AKT pathway was also involved in regulating the expression level of SHKBP1. Treatment of CD166^+^ OSCs with either an AKT1/2 kinase inhibitor (AKI, at 5 and 10 mM), a MEK inhibitor (U0126, at 5 and 10 mM), or PI3K inhibitor (LY294002, at 5 and 10 mM) decreased SHKBP1 expression in association with the specific inhibition of each targeted pathway (Additional file 1: Figure S2A).

By further define the role of SHKBP1 in EGFR activity regulation, we demonstrated that SHKBP1 knockdown in CD166^+^ OSCs resulted in an increase of EGFR phosphorylation in response to treatment with EGF (Additional file 1: Figure S2B). Furthermore, exposure to each inhibitor (LY294002, AKI, or U0126, at lower dose indicated above) increased the ratio of phospho-EGFR to EGFR upon ligand stimulation (Additional file 1: Figure S2C), consistent with the role of SHKBP1 in regulating EGFR activity. These data implies that TGFβ-induced activation of AKT co-opts an increased SHKBP1 expression, which further regulates EGFR activity.

### Overexpression of miR-499a inhibits TGFβ-induced resistance in vitro and in vivo

**Fig. 7:**
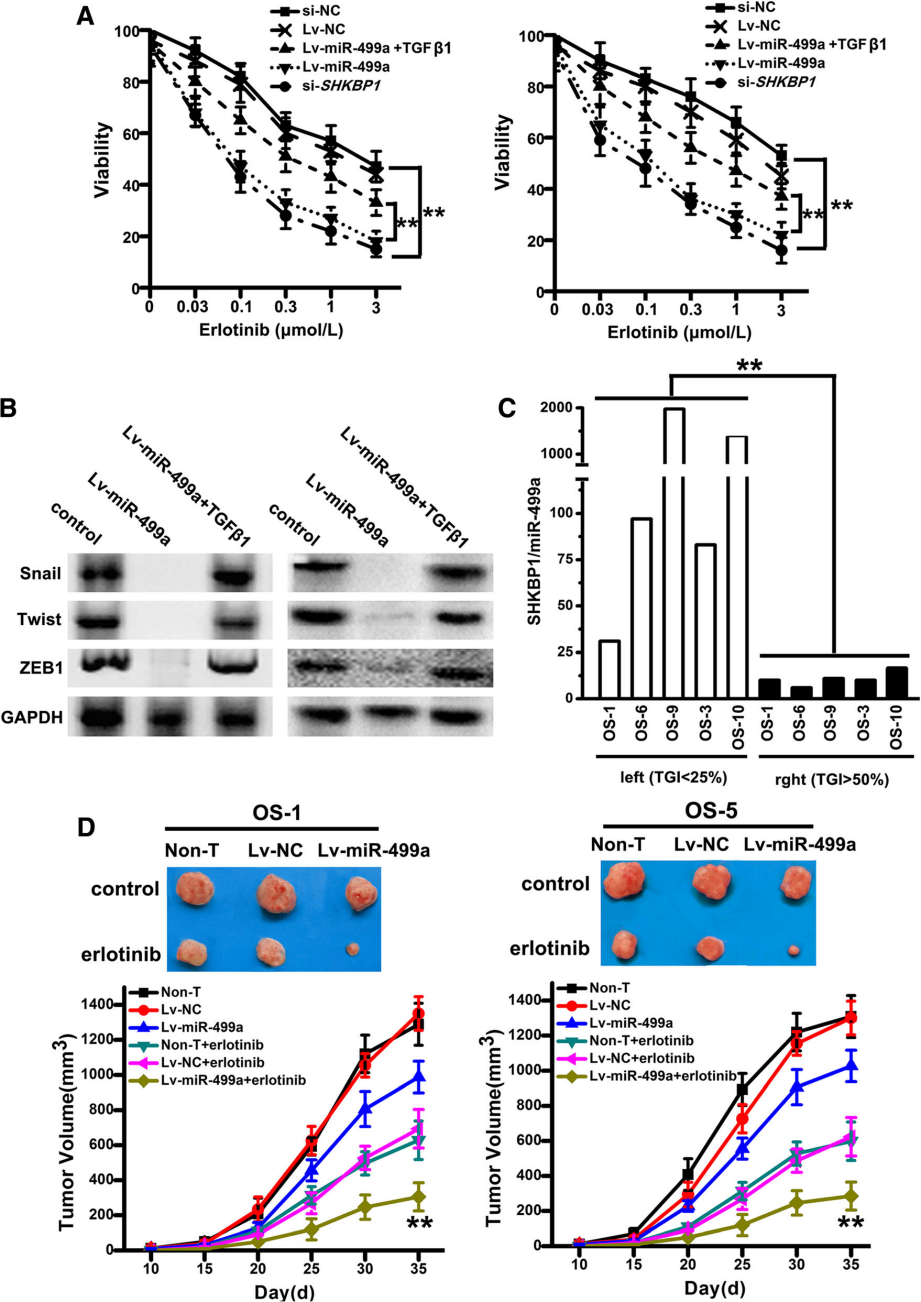
Overexpression of miR-499a inhibits TGFβ-induced resistance in vitro and in vivo*.* **a** With increasing concentrations of erlotinib from 0.03 to 3 μmol/L, the percentage of viable cells of CD166^+^ OSCs infected with Lv-miR-499a, Lv-NC, si-*SHKBP1* or si-NC were measured by MTT. Note: Columns, mean of three individual experiments; SD,**, *P* < 0.01. **b** Western-blotting was used with antibodies specific for Snail, Twist, and ZEB1 in CD166^+^ OSCs with or without infection of Lv-miR-499a. GAPDH was used as the control. **c** RNA was extracted from 5 OS directly xenografted tumors Levels of miR-499a were measured by qPCR. Note: Columns, mean of three individual experiments; SD,**, *P* < 0.01. **d** The potential of tumor initiation of CD166^+^ OSCs, Lv-NC*-*OSCs and si-*SHKBP1-* OSCs fractions by subcutaneous injection, and representative tumor volumes were measured following treatment with or without three cycles of erlotinib. Note: Columns, mean of three individual experiments; SD,**, *P* < 0.01

To further confirm whether miR-499a overexpression can inhibit TGFβ-induced erlotinib-resistance, a lentiviral-based approach was used to overexpress miR-499a: miR-499a mimics (Lv-miR-499a) or NC (Lv-NC). Also, SHKBP1 was knocked down by lentiviral-mediated si-*SHKBP1* (si-*SHKBP1*). With increasing concentrations of erlotinib, the percentage of viable cells in si-*SHKBP1*-OSCs and Lv-miR-499a-OSCs decreased more rapidly than Lv-NC and si-NC (Fig. 7a). Furthermore, Lv-miR-499a-OSCs showed reduced expression of EMT-related factors (Fig. 7b), however, the EMT-related factors were increased when Lv-miR-499a-OSCs were treated with TGF β1 (Fig. 7b).

In vivo assays, we obtained 5 human OS tissues that were directly xenografted into nude mice. We injected CD166^+^ OSCs clustered to the left and CD166^−^ cells clustered on the right. Relative tumor growth inhibition (TGI, the relative tumor growth of treated mice divided by relative tumor growth of control mice) in response to erlotinib (35 mg/kg) was calculated. Xenografts that display a high SHKBP1/miR-499a ratio tended to cluster on the left side of the chart, indicating that they were more resistant to erlotinib (Fig. 7c), which is in line with the clinic cohort results in Fig. 5c. We, next, conducted a tumorigenesis study to evaluate the role of miR-499a in erlotinib resistance of CD166^+^ OSCs. We injected CD166^+^ OSCs (non-T) and CD166^+^ OSCs infected with lentivirus mediated miR-499a mimics (Lv-miR-499a) or NC (Lv-NC) to build xenograft model on NOD-SCID mice. We found that xenograft in Lv-miR-499a group were slightly smaller (Fig. 7d), although no significant difference was observed. Upon intratumor injection with erlotinib, the size of xenograft in Lv-miR-499a group were significantly reduced (following the three cycles of treatment) in comparison with those in Lv-NC and CD166^+^ OSCs group (Fig. 7d).

## Discussion

Cancer stem cell-like cells (CSCs) have been identified in an increasing number of malignancies. Nevertheless, little is known about what regulates their critical ability to therapeutic resistance. CD166 has hardly been used as the cell-surface marker for sorting OSCs, even though Brune JC, et, al. [23] found that OS-derived mesenchymal stromal cells (MSC) showed normal MSC morphology and expressed the typical MSC surface marker profile (CD105/CD73/CD90/CD44/HLA-lassI/CD166 positive. Here, we found that the presence of a variable fraction of CD166^+^ cell populations in 10 out of 10 primary OS samples. These CD166^+^ cell populations were demonstrated not only bearing enhanced self-renewable and tumorigenic ability, but also resistant to erlotinib.

Erlotinib, a receptor TKI, is a drug used to treat non-small cell lung cancer, pancreatic cancer and several other types of cancer. It has been shown to be effective in patients with or without EGFR mutations [24, 25]. Certain mutations in the EGFR TK domain is one of the factors lead to the resistance to certain TKIs [14]. However, on the other hand, more and more studies showed that the genetic alterations are present in only a minority of patients who partially respond to treatment and are rare in tumors other than NSCLC [15, 26, 27]. Thus, the sensitivity of only a few tumors to EGFR TKIs can be explained by the presence of EGFR TK domain mutations. Actually, there are many other primary resistance mechanisms, include amplification of the MET gene [28], EMT [29], and cancer stem cell-like cells [30], contributing to the resistance to EGFR TKIs. In order to be able to provide treatment selectively to those patients who do not harbor EGFR mutations but will nonetheless respond to EGFR TKIs, there is an urgent need to define the precise molecular mechanisms underlying resistance to EGFR TKIs, and to identify specific biomarkers capable of predicting therapeutic response.

In OS, in vitro studies have reported the expression of EGFR as well as effective inhibition of OS growth by EGFR inhibitors [18, 19]. Here, we did not identify known mutations of TK domain from exons 18 to 21 that have been associated with constitutive EGFR activation in CD166^+^ cells from these 10 primary OS tissue samples, which is in line with the results of the former publications [24, 25].

TGFβs are believed to regulate tumor initiation and progression. According to the cancer type and tumor development timing, TGFβs act as both tumor suppressors and tumor promoters: however, it is now well accepted that TGFβs act as tumor promoters during the late stages of carcinogenesis to stimulate angiogenesis and immune evasion by inducing EMT. Previous studies have demonstrated that TGFβ plays a key role in promoting EMT driven by a network of transcriptional repressors that include SNAIL1, ZEB1, ZEB2, and TWIST [31]. In OS, studies demonstrated that TGFβs expression were enhanced, and TGFβs affected OS growth and lung metastatic development [9], suggesting that TGFβ plays a pro-tumoral effect in OS. Nevertheless, the downstream mechanism underlying TGFβ-induced EMT in OS is unclear. In this study, we found that, in CD166^+^ OSCs, TGFβ expression level was increased. TGFβ induced CD166^−^ cells to undergo an EMT-associated kinase switch that renders them resistant to EGFR TKIs, which suggests that increased autocrine exposure to TGFβ may be a driving force behind the erlotinib-resistant phenotype.

Accumulated studies uncovered that miR-499a was decreased in several cancers, including pancreatic cancer [32], hepatocellular carcinoma [33] and oral squamous cell carcinoma [34], functioning as the tumor suppressor. In OS, it was found inhibiting OS cell proliferation [35], however, the biological function and underlying mechanisms of miR-499a in OSCs was unclear. In this study, by characterizing the down-stream mechanisms of TGFβ-induced EMT in OS, we found that miR-499a was significantly reduced upon TGFβ treatment. Further mechanisms study defined that EMT factors, Snail1 and Zeb1, bound to the miR-499a promoters directly and regulated miR-499a at transcriptional level. As for its downstream signaling pathway, miR-499a was found targeting SHKBP1. Studies showed that compared with healthy individual’s serum, SHKBP1 protein expression in the serum of patients with lymph node metastasis and liver metastasis was significantly elevated [36], but studies on the regulating mechanisms SHKBP1 is few. Here, we unveiled that TGFβ-induced EMT in CD166^+^ OSCs results in decreased miR-499a and enhaced SHKBP1 expression level. As EGFR-independent activation of the PI3K-AKT pathway has frequently been seen in cells that develop resistance and is thought to confer resistance to EGFR TKIs [22], we further sought here that EMT-associated PI3K-AKT pathway co-opts to regulate the basal expression level of SHKBP1 in CD166^+^ OSCs. As for the downstream of SHKBP1, our data indicated that SHKBP1 regulated EGFR activity to lead to further EGFR TKIs resistance. Recently, Lifeng Feng, et, al., [37] found that SHKBP1 affected EGFR signaling that required at least one intact PXXXPR motif, which gives the insight of the possible molecular mechanisms of SHKBP1 in EGFR activity regulation. Therefore, TGFβ-mediated suppression of the miR-499a unleashes expression of SHKBP1, which in turn quenches EGFR activity. The elevation of SHKBP1 following TGFβ-induced EMT is sustained by EGFR-independent activation of AKT because this is reduced by PI3K inhibitors, but not by erlotinib.

In clinic, SHKBP1 expression level was found increased in OS samples compared with that in their adjacent normal counterparts. Meanwhile, SHKBP1 was negatively correlated with miR-499a level in CD166^+^ OSCs and corresponding CD166^−^ cells. Further xenografts assays showed that a high SHKBP1/miR-499a ratio tended to increased resistance to erlotinib. These results indicate that an elevated SHKBP1/miR-499a ratio may have clinical value as a predictive biomarker to characterize the erlotinib-resistant OS.

## Conclusion

**Fig. 8:**
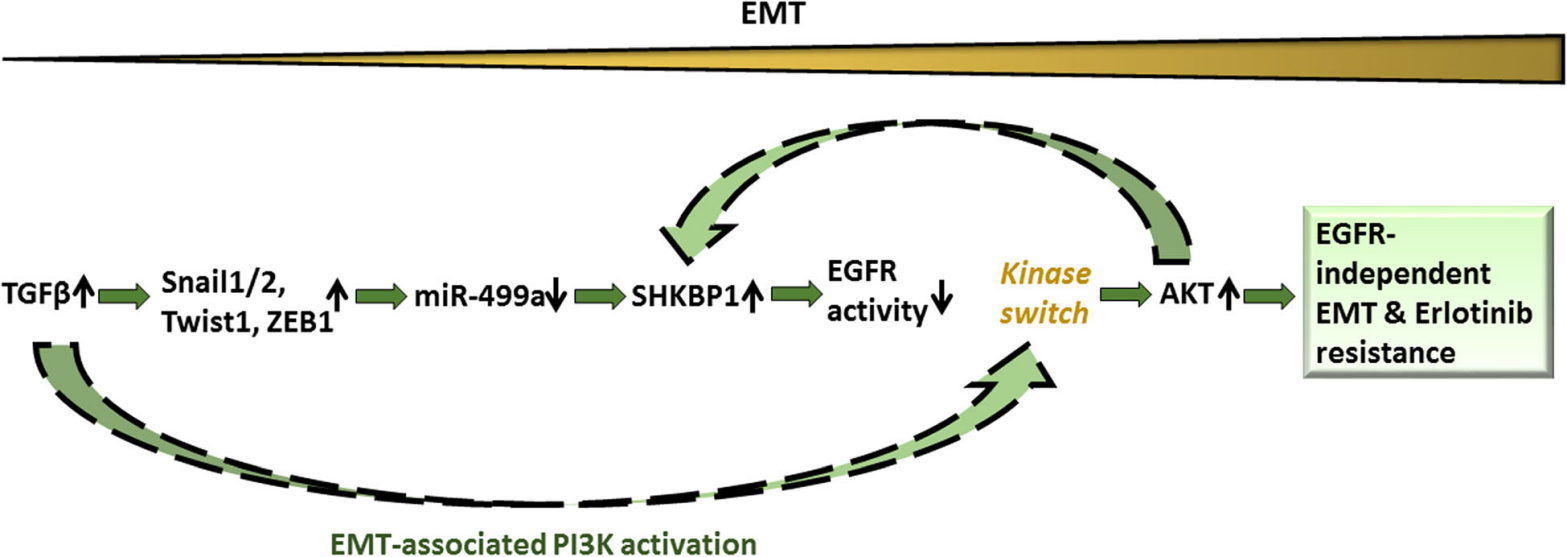
Evolution of erlotinib resistance in CD166^+^ OSCs

In conclusion, our data demonstrate that the TGFβ– miR-499a –SHKBP1 network orchestrates the EMT-associated kinase switch that induces resistance to EGFR TKIs in OSCs (Fig. 8). We also suggest that an elevated SHKBP1/miR-499a ratio is a molecular signature that characterizes the erlotinib-resistant OS, which may have clinical value as a predictive biomarker. Our data further suggest that inhibition of the molecular determinants of the EMT-associated kinase switch, here is TGFβ, may reverse the chemo-resistance of OSCs to EGFR inhibitors.

## Additional file

Additional file 1: **Table S1.** Osteosarcoma patients’ characteristics. **Figure S1.** miR-499a is directly regulated at transcriptional level by Snail1 and Zeb1. **Figure S2.** TGFβ-induced activation of AKT co-opts SHKBP1 that further regulates EGFR activity in CD166^+^ OSCs. (DOCX 394 kb)

## Abbreviations

ChIP: Chromatin immunoprecipitation
CSCs: Cancer stem cells
EGFR: Epidermal growth factor receptor
EMT: Epithelial-to-mesenchymal transition
FASC: Fluorescence-activated cell sorting
MSCs: Mesenchymal stromal cells
NSCLC: Non–small cell lung cancer
OS: Osteosarcoma
OSCs: Osteosarcoma stem cell-like cells
qPCR: quantitative real-time RT-PCR
TGFβ: Transforming growth factor beta
TK: Tyrosine kinase
TKIs: Tyrosine kinase inhibitors
