# Supplementary material for: The TGFβ-miR-499a-SHKBP1 pathway induces resistance to EGFR inhibitors in osteosarcoma cancer stem cell-like cells
Source: J Exp Clin Cancer Res. 2019 May 28;38:226. doi: 10.1186/s13046-019-1195-y (PMC6540516; doi:10.1186/s13046-019-1195-y)
Supplement: Supplementary file 1 — Table S1. Osteosarcoma patients’ characteristics. Figure S1. miR-499a is directly regulated at transcriptional level by Snail1 and Zeb1. Figure S2. TGFβ-induced activation of AKT co-opts SHKBP1 that further regulates EGFR activity in CD166+ OSCs. (DOCX 394 kb) [file 13046_2019_1195_MOESM1_ESM.docx]

**Additional file 1**

**Table S1. Osteosarcoma patients’ characteristics**

| **Case** | **Age** | **Gender** | **TNM** | **Clinical**  **stage** | **Sphere**  **formation** | **CD166 expression** | **^a^Normalized SHKBP1 amount 2^-ΔΔ Ct^** |
| --- | --- | --- | --- | --- | --- | --- | --- |
| OS-1 | 72 | Male | T1N0M0 | IA | Yes | 0.05% | 4.6372 |
| OS-2 | 54 | Male | T2N0M0 | IB | No | 0.01% | 3.9271 |
| OS-3 | 68 | Male | T1N1M0 | IIA | Yes | 0.15% | 5.2393 |
| OS-4 | 77 | Female | T2N2M0 | IIB | No | 0.09% | 6.1038 |
| OS-5 | 71 | Female | T3N0M0 | IIIA | Yes | 0.13% | 8.2766 |
| OS-6 | 59 | Male | T2N3M0 | IIIB | Yes | 0.24% | 6.0317 |
| OS-7 | 67 | Male | T2N3M0 | IIIB | Yes | 0.19% | 3.1297 |
| OS-8 | 81 | Female | T2N2M0 | IIIA | Yes | 0.07% | 4.3273 |
| OS-9 | 63 | Male | T3N3M1 | IV | Yes | 0.36% | 5.6916 |
| OS-10 | 73 | Female | T3N3M1 | IV | Yes | 0.47% | 4.3294 |

^a^ Relative quantification was performed by the 2^-ΔΔCt^ method with the adjacent normal tissue samples as a calibrator. Data show the means from three independent analyses. Every independent analysis was carried out after the RNA extraction step. Total RNA was poly-A tailed, reverse transcript, and then real-time PCR tested. ΔC_T_ obtained from real-time PCR was subject to paired t-test (ΔC_T_ = C_T_ _SHKBP1_-C_T β-actin_). The expression levels of SHKBP1 in osteosarcoma tumor tissues were significantly higher than adjacent normal tissues (P < 0.01).


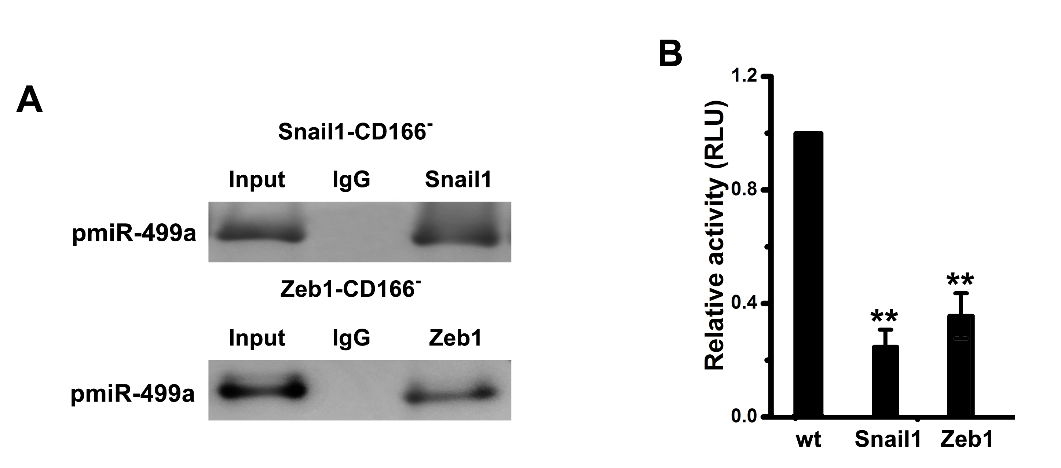


**Figure S1.** miR-499a is directly regulated at transcriptional level by Snail1 and Zeb1. (A) ChIP assays for miR-499a promoters in Snail1- or Zeb1- CD166^-^ cells, the input amount and control IgG are also shown. (B) The luciferase activity was measured, wt means wildtype. Note: Columns, mean of three individual experiments; SD,** P < 0.01.


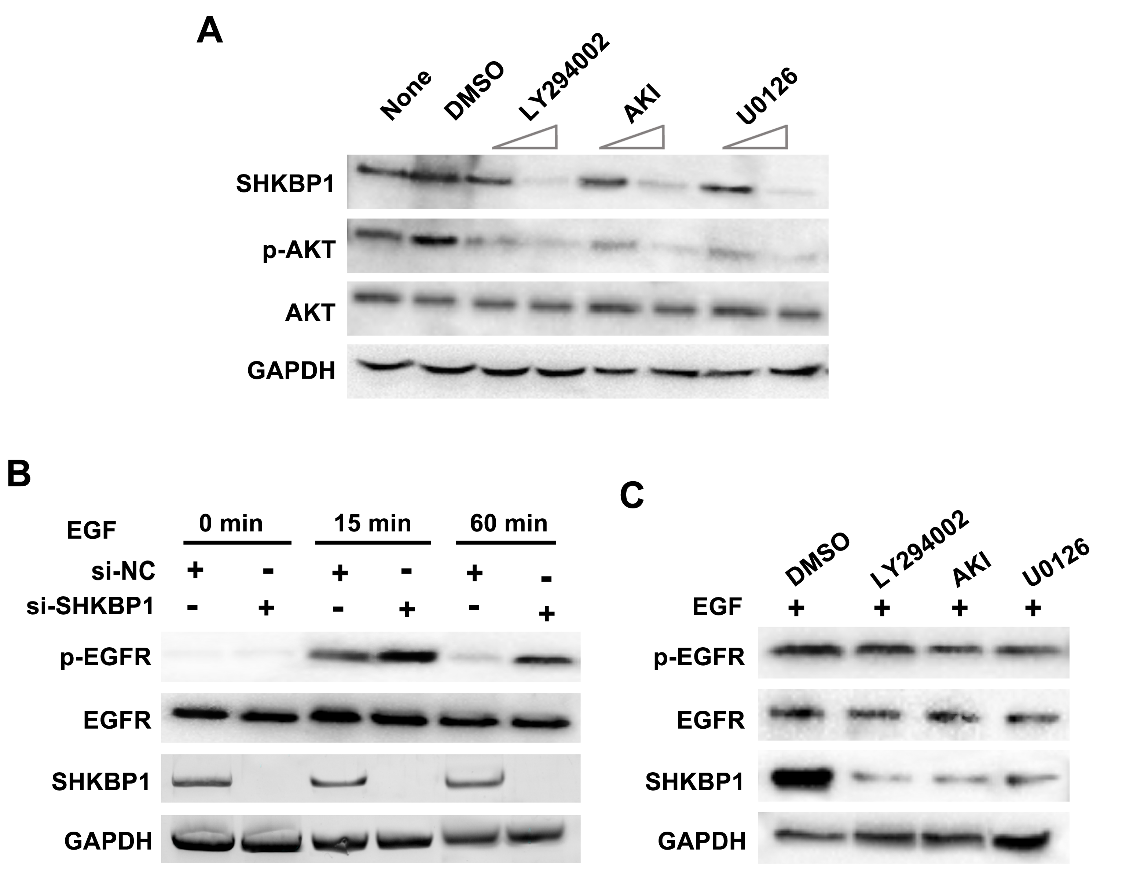


**Figure S2.** TGFβ-induced activation of AKT co-opts SHKBP1 that further regulates EGFR activity in CD166^+^ OSCs. (A) CD166^+^ OSCs were treated with LY294002 (PI3K inhibitor, at 5 or 10 mM), AKI (AKT1/2 kinase inhibitor, at 5 or 10 mM), U0126 (MEK1/2 inhibitor, at 5 or 10 mM), or DMSO (control) for 24 hrs and subjected to immunoblot analysis with indicated antibodies. GAPDH was used as the control. (B) CD166^+^ OSCs were transfected with either scrambled si-NC or siRNA targeting SHKBP1 (si-SHKBP1) for 48 hrs. Cells were stripped in serum free medium overnight and stimulated with EGF for 15 or 60 min. GAPDH was used as the control. (C) CD166^+^ OSCs were treated with 10 mM LY294002, 10 mM AKT1/2 kinase inhibitor, or 10 mM U0126 for 24 hrs. Cells were then treated with 10 ng/ml EGF for 30 min to induce EGFR phosphorylation and subjected to immunoblot analysis. GAPDH was used as the control.
